# Supplementary material for: Analysis of PPARγ Signaling Activity in Psoriasis
Source: Int J Mol Sci. 2021 Aug 10;22(16):8603. doi: 10.3390/ijms22168603 (PMC8395241; doi:10.3390/ijms22168603)
Supplement: Supplementary file 1 [file ijms-22-08603-s001.zip › Supplemental materials_Analysis of PPARg signaling activity in psoriasis/Pathway models/Models images and html files/Model of PPARG related pathways in psoriasis (short version)/11493.html]

TLR4


# Protein TLR4

|  |  |
| --- | --- |
| URN | urn:agi-llid:7099 |
| Total Entities | 0 |
| Connectivity | 9293 |
| Name | TLR4 |
| Description | toll-like receptor 4 |
| Notes | The protein encoded by this gene is a member of the Toll-like receptor (TLR) family which plays a fundamental role in pathogen recognition and activation of innate immunity. TLRs are highly conserved from Drosophila to humans and share structural and functional similarities. They recognize pathogen-associated molecular patterns that are expressed on infectious agents, and mediate the production of cytokines necessary for the development of effective immunity. The various TLRs exhibit different patterns of expression. This receptor has been implicated in signal transduction events induced by lipopolysaccharide (LPS) found in most gram-negative bacteria. Mutations in this gene have been associated with differences in LPS responsiveness. Multiple transcript variants encoding different isoforms have been found for this gene. [provided by RefSeq, Jan 2012] |
| Primary Cell Localization | Plasma membrane |
| Class | Receptor |
| ObjectType | Protein |

---

|  |  |
| --- | --- |
| Pathway | I-cell: CCK-8 Secretion and Regulation of Eating Behavior |
|  | Cortisol in Resolving Inflammation |
|  | Antiviral Signaling through Pattern Recognition Receptors |
|  | DC-SIGN (CD209) Signaling |
|  | DCIR1 (CLEC4A) Signaling |
|  | TLR4 Signaling in Leukocytes |
|  | T-Cell Independent B-Cell Activation |
|  | Toll-like Receptors in Sterile Inflammation |
|  | Necrosis |
|  | MC1R in anti-Inflammatory Signaling |
|  | CD16/CD14 Proinflammatory Monocyte Activation |
|  | AHR Signaling in M1 Macrophages Function |
|  | AXL Receptor Inhibits Macrophages and Dendritic Cells Function |
|  | Neutrophil Recruitment and Priming |
|  | Positive Acute Phase Proteins Synthesis |
|  | Vascular Endothelial Cell Activation by Blood Coagulation Factors |
|  | Adipokines Production by Adipocyte |
|  | Monoblast -> Promonocyte Surface Expression Markers |
|  | Myeloblast -> Neutrophil Surface Expression Markers |
|  | Proteins Involved in Atherosclerosis |
|  | Dendritic Cells Function in Atherosclerosis |
|  | RAGE/AGER and S100 Proteins in Cardiovascular Injury |
|  | Low-Density Lipoproteins and Chemokines in Atherosclerosis |
|  | Proteins Involved in Dilated Cardiomyopathy |
|  | Proteins Involved in Pulmonary Hypertension |
|  | Myocardial Infarction |
|  | Proteins Involved in Myocardial Ischemia |
|  | Proteins Involved in Myocarditis |
|  | Toll-like Receptors (TLR) in Antiviral Innate Immune Response |
|  | Proteins Involved in Acne Vulgaris |
|  | Dendritic Cells Function in Psoriasis |
|  | Apoptotic Keratinocytes Clearance Recession in Systemic Lupus Erythematosus |
|  | Dendritic Cell Activation in Systemic Lupus Erythematosis |
|  | Proteins Involved in Systemic Lupus Erythematosus |
|  | Plasmacytoid Dendritic Cell in Diabetes Mellitus Type 1 |
|  | TLR2 Signaling in Treg-Cell in Type 1 Diabetes (Animal Model) |
|  | Toll-like Receptors in beta-Cell |
|  | Proteins Involved in Diabetes Mellitus Type 1 |
|  | Conventional Dendritic Cell Role in Diabetes Mellitus Type 1 |
|  | Proteins Involved in Diabetes Mellitus Type 2 |
|  | Proteins Involved in Diabetic Nephropathy |
|  | Proteins Involved in Diabetic Neuropathy |
|  | Proteins Involved in Insulin Resistance |
|  | Insulin Resistance in Myocytes Induced by Obesity |
|  | Hepatic Stellate Cells in non-Alcoholic Fatty Liver Disease |
|  | Proteins Involved in non-Alcoholic Fatty Liver Disease |
|  | Thymic Follicular Hyperplasia |
|  | Adipokines Production by Adipocyte Impaired in Obesity |
|  | Proteins Involved in Obesity |
|  | Proteins Involved in Celiac Disease |
|  | Celiac Disease |
|  | Proteins Involved in Colorectal Neoplasms |
|  | Crohn's Disease |
|  | Paneth-Cell Function in Crohn's Disease |
|  | Proteins Involved in Inflammatory Bowel Diseases |
|  | Dendritic Cell Dysfunction in Crohn's disease |
|  | Proteins Involved in Helicobacter Infections |
|  | Dendritic Cell Function in Ulcerative Colitis |
|  | Epithelial Cell in the Innate Immune Response in Ulcerative Colitis |
|  | Nicotine anti-Inflammatory Effect in Ulcerative Colitis |
|  | Proteins Involved in Ulcerative Colitis |
|  | Antiphospholipid Antibodies in Endothelial Cell |
|  | Proteins Involved in Antiphospholipid Syndrome |
|  | B-Cell Chronic Lymphocytic Leukemia |
|  | Proteins Involved in Marginal Zone Lymphoma |
|  | Antiphospholipid Antibodies and Endothelial Cell Interaction |
|  | Club-Cell Role in Asthma |
|  | Antigen-Presenting Cell Role in Asthma |
|  | Mucin Production in Goblet Airway Epithelial Cells |
|  | Proteins Involved in Cystic Fibrosis |
|  | Airway Epithelial Cell Dysfunction |
|  | CFTR Related Ion-Channel Dysfunction in Cystic Fibrosis Airway Epithelium (Class III Mutations) |
|  | Proteins Involved in Gout |
|  | Monocytes Function in Gout |
|  | Proteins Involved in Osteoarthritis |
|  | Dendritic Cell Dysfunction in Psoriatic Arthritis |
|  | TLR2 Induced Synovial Fibroblast Activation in Rheumatoid Arthritis |
|  | Proteins Involved in Rheumatoid Arthritis |
|  | Proteins Involved in HIV Type 1 Infection |
|  | Toll-like Receptors (TLR) in Periodontitis |
|  | Proteins Involved in Periodontitis |
|  | IL1 Receptor Antagonist (IL1RN) Deficiency (DIRA) |
|  | PAPA Syndrome |
|  | TNF Receptor Associated Periodic Syndrome |
|  | Mevalonate Kinase Deficiency |
|  | Blau Syndrome |
|  | Cryopyrin (NLPR3) Associated Periodic Syndromes |
|  | Familial Cold Autoinflammatory Syndrome 2 |
|  | Familial Mediterranean Fever |
|  | Majeed Syndrome |
|  | Gluten Impact on Neuronal System (Hypothesis) |
|  | Microglia Activation in Alzheimer's Disease |
|  | Metals and Amyloid beta Toxicity in Alzheimer's Disease |
|  | HMGB1 and IL1B in Neuroinflammation |
|  | HMGB1 and IL1B in Neuronal Hyperexcitation |
|  | Microglia Activation in Parkinson's Disease |
|  | Proteins Involved in Parkinson's Disease |
|  | Proteins Involved in Amyotrophic Lateral Sclerosis |
|  | Proteins Involved in Endometriosis |
|  | Proteins Involved in Spontaneous Abortion |
|  | Proteins Involved in Cholesteatoma |
|  | Cholesteatoma |
|  | Proteins Involved in HPV Infection |
|  | HPV E6 and E7 Induced Disruption of TNFR/NF-kB Signaling in Keratinocytes |
|  | Proteins Involved in Age-Related Macular Degeneration |
|  | Genes with Mutations Associated with Age-Related Macular Degeneration |
|  | Proteins Involved in Hearing Loss |
|  | MUC5AC Expression by NTHi and S. Pneumoniae |
|  | Immune System Role in Otitis Media |
|  | Alveolar Macrophage Negative Regulation Declined in COPD |
|  | Proteins with Altered Expression in Chronic Obstructive Pulmonary Disease |
|  | Alveolar Macrophages Dysfunction in COPD |
|  | Proteins Involved in Chronic Obstructive Pulmonary Disease |
|  | T-Cell Cytotoxic Activation |
|  | Alveolar Epithelial Cell Dysfunction in COPD |
|  | Proteins Involved in Pulmonary Emphysema |
|  | Mucin Hyperproduction in Goblet and Mucous Cells |
|  | Proteins Involved in Glomerulonephritis |
|  | Endothelial Cell Dysfunction in Glomerulonephritis |
|  | Toll-like Receptors in Pyelonephritis |
|  | CSF2 -> NF-kB Expression Targets |
|  | NFKB Canonical Signaling Activation in Cancer |
|  | NFKB Signaling Activation by Blocking of Tumor Suppressors |
|  | IDO1 in Cancer Immune Escape |
|  | IFNG/IFNR Expression Targets |
|  | CD Markers -> Expression Targets in Lymphoid System and Blood |
|  | GPCRs Family -> Expression Targets in Lymphoid System and Blood |
|  | Elevated Receptors -> Expression Targets in Brain |
|  | AGT -> CREB Expression Targets |
|  | NF-kB Canonical Signaling |
|  | TLR4 -> AP-1 Expression Targets |
|  | TLR5 Expression Targets |
|  | TLR4 -> AP-1/EGR1/HIF1A Expression Targets |
|  | TLR1 -> 2/6 Expression Targets |
|  | TLR4 -> NF-kB/IRF Expression Targets |
|  | TLR9 Expression Targets |
|  | TLR4 -> IRF Signaling |
|  | TLR4/5/7/9 -> NF-kB Signaling |
|  | TLR -> AP-1 Signaling |
|  | pathway 4 |
|  | prarg negative regulators, ps-positive |
|  | pprarg neg, uknown targets, ps-positive |
|  | prarg neg,ukn expres targets, ps-positive |
|  | PPARG negative regulators |
|  | PPARG negative targets |
|  | pparg expr target\_regulators |
|  | Neighbors of disease exacerbation |
|  | Neighbors of tumor progression |
|  | Neighbors of cancer progression |
|  | Neighbors of inflammatory status |
|  | Neighbors of overall survival |
|  | Neighbors of disease severity |
|  | Neighbors of tumor size |
|  | Neighbors of lesion size |
|  | Neighbors of mortality |
|  | Neighbors of survival rate |
|  | Neighbors of infarct size |
|  | Neighbors of survival time |
|  | Neighbors of functional recovery |
|  | Neighbors of insulin sensitivity |
|  | Neighbors of bacterial load |
|  | Neighbors of cancer survival |
|  | Neighbors of graft survival |
|  | Neighbors of therapeutic efficacy |
|  | Neighbors of clinical stage |
|  | Neighbors of lethality |
|  | Neighbors of tumor regression |
|  | Neighbors of disease-free survival |
|  | Neighbors of tumor response |
|  | Neighbors of macrophage count |
|  | Neighbors of long-term survival |
|  | Neighbors of treatment outcome |
|  | Neighbors of bone mass |
|  | Neighbors of body weight |
|  | Neighbors of body weight gain |
|  | Neighbors of cardiovascular risk |
|  | Neighbors of tumor recurrence |
|  | Neighbors of tumor promotion |
|  | Neighbors of neutrophil count |
|  | Neighbors of glucose tolerance |
|  | Neighbors of morbidity |
|  | Neighbors of fibrosis progression |
|  | Neighbors of fat mass |
|  | Neighbors of exhaustion |
|  | Neighbors of bone mineral density |
|  | Neighbors of adverse outcome |
|  | Neighbors of radioresistance |
|  | Neighbors of lesion area |
|  | Neighbors of cancer-specific survival |
|  | Neighbors of progression-free survival |
|  | Neighbors of apoptosis rate |
|  | Neighbors of shear stress |
|  | Neighbors of viral load |
|  | Neighbors of blood pressure |
|  | Neighbors of leukocyte count |
|  | Neighbors of tumor grade |
|  | Neighbors of vascular reactivity |
|  | Neighbors of vascular leakage |
|  | Neighbors of vascular density |
|  | Neighbors of cancer risk |
|  | Neighbors of cancer incidence |
|  | Neighbors of tumor aggressiveness |
|  | Neighbors of systolic blood pressure |
|  | Neighbors of tumor resistance |
|  | Neighbors of blood glucose |
|  | Neighbors of ejection fraction |
|  | Neighbors of median survival |
|  | Neighbors of renal hypertrophy |
|  | Neighbors of fasting plasma glucose |
|  | Neighbors of T-cell count |
|  | Neighbors of antimetastatic activity |
|  | Neighbors of energy expenditure |
|  | Neighbors of glucose level |
|  | Neighbors of radiosensitivity |
|  | Neighbors of left ventricular ejection fraction |
|  | Neighbors of pregnancy outcome |
|  | Neighbors of gastric cancer risk |
|  | Neighbors of neurological outcome |
|  | Neighbors of infarct area |
|  | Neighbors of fertility |
|  | Neighbors of recurrence rate |
|  | Neighbors of pathological response |
|  | Neighbors of lymphocyte count |
|  | Neighbors of metabolic response |
|  | Neighbors of insulin level |
|  | Neighbors of comorbidity |
|  | Neighbors of fibroblast phenotype |
|  | Neighbors of disease exacerbation |
|  | Neighbors of tumor progression |
|  | Neighbors of cancer progression |
|  | Neighbors of inflammatory status |
|  | Neighbors of overall survival |
|  | Neighbors of disease severity |
|  | Neighbors of tumor size |
|  | Neighbors of lesion size |
|  | Neighbors of mortality |
|  | Neighbors of survival rate |
|  | Neighbors of infarct size |
|  | Neighbors of survival time |
|  | Neighbors of functional recovery |
|  | Neighbors of insulin sensitivity |
|  | Neighbors of bacterial load |
|  | Neighbors of cancer survival |
|  | Neighbors of graft survival |
|  | Neighbors of therapeutic efficacy |
|  | Neighbors of clinical stage |
|  | Neighbors of lethality |
|  | Neighbors of tumor regression |
|  | Neighbors of disease-free survival |
|  | Neighbors of tumor response |
|  | Neighbors of macrophage count |
|  | Neighbors of long-term survival |
|  | Neighbors of treatment outcome |
|  | Neighbors of bone mass |
|  | Neighbors of body weight |
|  | Neighbors of body weight gain |
|  | Neighbors of cardiovascular risk |
|  | Neighbors of tumor recurrence |
|  | Neighbors of tumor promotion |
|  | Neighbors of neutrophil count |
|  | Neighbors of glucose tolerance |
|  | Neighbors of morbidity |
|  | Neighbors of fibrosis progression |
|  | Neighbors of fat mass |
|  | Neighbors of exhaustion |
|  | Neighbors of bone mineral density |
|  | Neighbors of adverse outcome |
|  | Neighbors of radioresistance |
|  | Neighbors of lesion area |
|  | Neighbors of cancer-specific survival |
|  | Neighbors of progression-free survival |
|  | Neighbors of apoptosis rate |
|  | Neighbors of shear stress |
|  | Neighbors of viral load |
|  | Neighbors of blood pressure |
|  | Neighbors of leukocyte count |
|  | Neighbors of tumor grade |
|  | Neighbors of vascular reactivity |
|  | Neighbors of vascular leakage |
|  | Neighbors of vascular density |
|  | Neighbors of cancer risk |
|  | Neighbors of cancer incidence |
|  | Neighbors of tumor aggressiveness |
|  | Neighbors of systolic blood pressure |
|  | Neighbors of tumor resistance |
|  | Neighbors of blood glucose |
|  | Neighbors of ejection fraction |
|  | Neighbors of median survival |
|  | Neighbors of renal hypertrophy |
|  | Neighbors of fasting plasma glucose |
|  | Neighbors of T-cell count |
|  | Neighbors of antimetastatic activity |
|  | Neighbors of energy expenditure |
|  | Neighbors of glucose level |
|  | Neighbors of radiosensitivity |
|  | Neighbors of left ventricular ejection fraction |
|  | Neighbors of pregnancy outcome |
|  | Neighbors of gastric cancer risk |
|  | Neighbors of neurological outcome |
|  | Neighbors of infarct area |
|  | Neighbors of fertility |
|  | Neighbors of recurrence rate |
|  | Neighbors of pathological response |
|  | Neighbors of lymphocyte count |
|  | Neighbors of metabolic response |
|  | Neighbors of insulin level |
|  | Neighbors of comorbidity |
|  | Neighbors of fibroblast phenotype |
|  | Neighbors of cell development |
|  | Neighbors of immune response |
|  | Neighbors of cell population |
|  | Neighbors of T-cell development |
|  | Neighbors of inflammatory response |
|  | Neighbors of wound healing |
|  | Neighbors of angiogenesis |
|  | Neighbors of innate immune response |
|  | Neighbors of cell function |
|  | Neighbors of macrophage activation |
|  | Neighbors of cell formation |
|  | Neighbors of adaptive immune response |
|  | Neighbors of osteoclast development |
|  | Neighbors of epithelial to mesenchymal transition |
|  | Neighbors of immunity |
|  | Neighbors of chemotaxis |
|  | Neighbors of dendritic cell differentiation |
|  | Neighbors of cell survival |
|  | Neighbors of osteoclast differentiation |
|  | Neighbors of cell phenotype |
|  | Neighbors of T-cell proliferation |
|  | Neighbors of tissue remodeling |
|  | Neighbors of cell adhesion |
|  | Neighbors of T-cell activation |
|  | Neighbors of endothelial cell proliferation |
|  | Neighbors of fibrogenesis |
|  | Neighbors of tumor growth |
|  | Neighbors of dendritic cell development |
|  | Neighbors of tissue repair |
|  | Neighbors of cell count |
|  | Neighbors of cell infiltration |
|  | Neighbors of cell interaction |
|  | Neighbors of SMC proliferation |
|  | Neighbors of lumen formation |
|  | Neighbors of endothelial cell migration |
|  | Neighbors of adipogenesis |
|  | Neighbors of lipid storage |
|  | Neighbors of cell damage |
|  | Neighbors of pregnancy |
|  | Neighbors of monocyte differentiation |
|  | Neighbors of aging |
|  | Neighbors of cell motility |
|  | Neighbors of bone resorption |
|  | Neighbors of monocyte migration |
|  | Neighbors of fibroblast proliferation |
|  | Neighbors of adipocyte differentiation |
|  | Neighbors of macrophage migration |
|  | Neighbors of microglial activation |
|  | Neighbors of neutrophil recruitment |
|  | Neighbors of regeneration |
|  | Neighbors of cell migration |
|  | Neighbors of cancer growth |
|  | Neighbors of stem cell proliferation |
|  | Neighbors of smooth muscle cell migration |
|  | Neighbors of hepatic regeneration |
|  | Neighbors of transendothelial migration |
|  | Neighbors of phagocytosis |
|  | Neighbors of immune system activation |
|  | Neighbors of leukocyte migration |
|  | Neighbors of leukocyte recruitment |
|  | Neighbors of osteoclast formation |
|  | Neighbors of monocyte recruitment |
|  | Neighbors of cell invasion |
|  | Neighbors of heart function |
|  | Neighbors of hemopoiesis |
|  | Neighbors of bone remodeling |
|  | Neighbors of ossification |
|  | Neighbors of T-cell function |
|  | Neighbors of immune system function |
|  | Neighbors of lung development |
|  | Neighbors of autophagy |
|  | Neighbors of monocyte adhesion |
|  | Neighbors of transcription activation |
|  | Neighbors of senescence |
|  | Neighbors of T-cell response |
|  | Neighbors of tumor immunity |
|  | Neighbors of hepatocyte proliferation |
|  | Neighbors of cell transdifferentiation |
|  | Neighbors of neuroprotection |
|  | Neighbors of stem cell differentiation |
|  | Neighbors of endothelial cell function |
|  | Neighbors of neuronal death |
|  | Neighbors of cell growth |
|  | Neighbors of ROS generation |
|  | Neighbors of epithelial cell proliferation |
|  | Neighbors of first trimester pregnancy |
|  | Neighbors of cancer cell growth |
|  | Neighbors of sensitization |
|  | Neighbors of hemato-encephalic barrier |
|  | Neighbors of macrophage function |
|  | Neighbors of cellular immune response |
|  | Neighbors of cellular senescence |
|  | Neighbors of colony formation |
|  | Neighbors of macrophage differentiation |
|  | Neighbors of cell differentiation |
|  | Neighbors of keratinocyte proliferation |
|  | Neighbors of stem cell migration |
|  | Neighbors of life span |
|  | Neighbors of cell homeostasis |
|  | Neighbors of psoriasis |
|  | Neighbors of inflammatory disease |
|  | Neighbors of rheumatoid arthritis |
|  | Neighbors of atherosclerosis |
|  | Neighbors of fibrosis |
|  | Neighbors of colitis |
|  | Neighbors of arthritis |
|  | Neighbors of inflammatory bowel disease |
|  | Neighbors of injury |
|  | Neighbors of atherogenesis |
|  | Neighbors of vascular remodeling |
|  | Neighbors of metastasis |
|  | Neighbors of carcinogenesis |
|  | Neighbors of multiple sclerosis |
|  | Neighbors of chronic inflammation |
|  | Neighbors of inflammation |
|  | Neighbors of tumor microenvironment |
|  | Neighbors of pathological angiogenesis |
|  | Neighbors of obesity |
|  | Neighbors of vascular disease |
|  | Neighbors of diabetes mellitus |
|  | Neighbors of asthma |
|  | Neighbors of pneumonia |
|  | Neighbors of hyperplasia |
|  | Neighbors of osteoarthritis |
|  | Neighbors of liver fibrosis |
|  | Neighbors of chronic obstructive pulmonary disease |
|  | Neighbors of myocardial infarction |
|  | Neighbors of leukocyte infiltration |
|  | Neighbors of reperfusion injury |
|  | Neighbors of airway inflammation |
|  | Neighbors of hepatitis |
|  | Neighbors of insulin resistance |
|  | Neighbors of infection |
|  | Neighbors of vascular injury |
|  | Neighbors of renal injury |
|  | Neighbors of preeclampsia |
|  | Neighbors of macrophage infiltration |
|  | Neighbors of liver injury |
|  | Neighbors of neuroinflammation |
|  | Neighbors of sepsis |
|  | Neighbors of death |
|  | Neighbors of type 1 diabetes |
|  | Neighbors of encephalomyelitis |
|  | Neighbors of autoimmune disease |
|  | Neighbors of ischemic stroke |
|  | Neighbors of dermatitis |
|  | Neighbors of neutrophil infiltration |
|  | Neighbors of heart disease |
|  | Neighbors of diabetic nephropathy |
|  | Neighbors of lung injury |
|  | Neighbors of immunopathology |
|  | Neighbors of systemic sclerosis |
|  | Neighbors of hepatocellular carcinoma |
|  | Neighbors of autoimmunity |
|  | Neighbors of acute lung injury |
|  | Neighbors of gastric cancer |
|  | Neighbors of atherosclerotic plaque |
|  | Neighbors of experimental autoimmune encephalomyelitis |
|  | Neighbors of colorectal cancer |
|  | Neighbors of cardiac remodeling |
|  | Neighbors of breast cancer |
|  | Neighbors of ischemia |
|  | Neighbors of systemic lupus erythematosus |
|  | Neighbors of melanoma |
|  | Neighbors of collagen-induced arthritis |
|  | Neighbors of cartilage degeneration |
|  | Neighbors of interstitial fibrosis |
|  | Neighbors of neoplasm invasion |
|  | Neighbors of kidney disease |
|  | Neighbors of heart failure |
|  | Neighbors of lung disease |
|  | Neighbors of pulmonary fibrosis |
|  | Neighbors of cardiovascular disease |
|  | Neighbors of stroke |
|  | Neighbors of cancer |
|  | Neighbors of cerebral ischemia |
|  | Neighbors of type 2 diabetes |
|  | Neighbors of neoplasm |
|  | Neighbors of endometriosis |
|  | Neighbors of neointima |
|  | Neighbors of vasculitis |
|  | Neighbors of atrophy |
|  | Neighbors of diet-induced obesity |
|  | Neighbors of respiratory hypersensitivity |
|  | Neighbors of myocardial fibrosis |
|  | Neighbors of synovitis |
|  | Neighbors of coronary artery disease |
|  | Neighbors of rupture |
|  | Neighbors of endothelial cell dysfunction |
|  | Neighbors of liver disease |
|  | Neighbors of prostate cancer |
|  | Neighbors of hypertrophy |
|  | Neighbors of osteolysis |
|  | Neighbors of ovarian cancer |
|  | Neighbors of pancreatic cancer |
|  | Neighbors of hypertension |
|  | Neighbors of intimal hyperplasia |
|  | Neighbors of allograft rejection |
|  | Neighbors of proteinuria |
|  | Neighbors of epithelial cell |
|  | Neighbors of keratinocyte |
|  | Neighbors of vascular smooth muscle cell |
|  | Neighbors of monocyte |
|  | Neighbors of macrophage |
|  | Neighbors of adipocyte |
|  | Neighbors of fibroblast |
|  | Neighbors of airway epithelial cell |
|  | Neighbors of PBMC |
|  | Neighbors of microglia |
|  | Neighbors of hepatocyte |
|  | Neighbors of kidney mesangial cell |
|  | Neighbors of endothelial cell |
|  | Neighbors of dendritic cell |
|  | Neighbors of Kuppfer cell |
|  | Neighbors of proadipocyte |
|  | Neighbors of kidney tubule cell |
|  | Neighbors of inflammatory cell |
|  | Neighbors of bone marrow macrophage |
|  | Neighbors of neutrophil |
|  | Neighbors of astrocyte |
|  | Neighbors of bone marrow derived dendritic cell |
|  | Neighbors of immunocompetent cell |
|  | Neighbors of granulocyte |
|  | Neighbors of cardiac myocyte |
|  | Neighbors of stem cell |
|  | Neighbors of B-cell |
|  | Neighbors of leukocyte |
|  | Neighbors of T-cell |
|  | Neighbors of mast cell |
|  | Neighbors of neuroglia |
|  | Neighbors of podocyte |
|  | Neighbors of conventional dendritic cell |
|  | Neighbors of phagocyte |
|  | Neighbors of kidney cell |
|  | Neighbors of lung |
|  | Neighbors of liver |
|  | Neighbors of kidney |
|  | Neighbors of intestine |
|  | Neighbors of lymph node |
|  | Neighbors of colon |
|  | Neighbors of airway |
|  | Neighbors of heart |
|  | Neighbors of gastrointestinal tract |
|  | Neighbors of placenta |
|  | Neighbors of synovial membrane |
|  | Neighbors of uterus |
|  | Neighbors of periodontium |
|  | Neighbors of retina |
|  | Neighbors of brain |
|  | Neighbors of stomach |
|  | Neighbors of islets of Langerhans |
|  | Neighbors of artery |
|  | Neighbors of cardiovascular system |
|  | Neighbors of prostate |
|  | Neighbors of central nervous system |
|  | Neighbors of kidney glomerulus |
|  | Neighbors of immune system |
|  | Neighbors of left ventricle |
|  | Neighbors of uterine cervix |
|  | Neighbors of omentum |
|  | Neighbors of arteriole |
|  | Neighbors of bronchus |
|  | Neighbors of glands |
|  | Neighbors of eye |
|  | Neighbors of mammary gland |
|  | Neighbors of thymus gland |
|  | Neighbors of nasopharynx |
|  | Neighbors of lower extremity |
|  | Neighbors of small intestine |
|  | Neighbors of amniotic cavity |
|  | Neighbors of NF-kB family |
|  | Neighbors of oxidized LDL |
|  | Neighbors of JNK |
|  | Neighbors of PPAR |
|  | Neighbors of Jun/Fos |
|  | Neighbors of NFKBI |
|  | Neighbors of PKC |
|  | Neighbors of PI3K |
|  | Neighbors of STAT family |
|  | Neighbors of mitogen-activated protein kinase |
|  | Neighbors of transforming growth factor |
|  | Neighbors of TLR |
|  | Neighbors of cytokine |
|  | Neighbors of ERK1/2 |
|  | Neighbors of PKA |
|  | Neighbors of ovalbumin |
|  | Neighbors of LDL |
|  | Neighbors of NF-AT family |
|  | Neighbors of adipokine |
|  | Neighbors of NAD(P)H oxidase |
|  | Neighbors of IgG |
|  | Neighbors of AMPK |
|  | Neighbors of LXR |
|  | Neighbors of HDL |
|  | Neighbors of inflammatory cytokine |
|  | Neighbors of histone deacetylase |
|  | Neighbors of PDGF-BB |
|  | Neighbors of HIF-1 |
|  | Neighbors of GPCR |
|  | Neighbors of Notch |
|  | Neighbors of heat shock protein 90 |
|  | Neighbors of lectin |
|  | Neighbors of fibrinogen |
|  | Neighbors of aldehyde reductase |
|  | Neighbors of Ang II receptor |
|  | Neighbors of non-coding RNA |
|  | Neighbors of histone H3 |
|  | Neighbors of retinoid-X receptor subfamily |
|  | Neighbors of immunoglobulin |
|  | Neighbors of TLR1/2 heterodimer |
|  | Neighbors of angiotensin receptor |
|  | Neighbors of histone |
|  | Neighbors of neuropeptides |
|  | Neighbors of pancreatic elastase |
|  | Neighbors of phosphoinositide phospholipase C |
|  | Neighbors of TLR4/Myd88 |
|  | Neighbors of TNF-alpha receptor |
|  | Model of PPARG signaling in psoriasis |
|  | PPARG negative regulators and targets |
|  | Model of PPARG related pathways in psoriasis (short version) |
|  | Vg9Vd2 cells-\|SARS |
|  | AGER feed forward loop in innate immunity |
|  | Dendritic Cell Activation in COVID19 |
|  | Overview. Diseases of the digestive system (chapter 10) |
|  | Overview. Diseases of the eye (chapter 6) |
|  | Overview. Diseases of the ear (chapter 7) |
|  | Overview. Endocrine and metabolic diseases (chapter 4) |
|  | 1\_Inflammation in keratinocytes in the pathology of acne |
|  | 1\_The role of Toll-like receptors in pyelonephritis |
|  | 1\_Insufficient activation of immune response in the middle ear epithelium cells in otitis media |
|  | 2\_Pathogens stimulate mucins expression in the middle ear |
|  | 1\_Low-density lipoprotein internalization and the accumulation of lipids in atherosclerosis |
|  | 3\_Arterial wall inflammation and calcification in atherosclerosis |
|  | 1\_Cigarette smoke causes dysfunction and production of inflammatory mediators in alveolar epithelial cells |
|  | 3\_Role of alveolar macrophages in pulmonary tissue destruction in COPD |
|  | 1\_Th2 cell response in asthma |
|  | 3\_1\_Mucus accumulation in asthma |
|  | 4\_Mucin hyperproduction in goblet and mucous cells in COPD |
|  | 2\_Paneth cell dysfunction in Crohn's disease |
|  | 1\_Defects in response to pathogens in the gut promotes inflammation in Crohn's disease |
|  | 2\_Intestine epithelial cells drive immunological responses in ulcerative colitis |
|  | 1\_Intestine epithelial cell dysfunction in ulcerative colitis |
|  | 4\_1\_Polymorphisms associated with inflammatory bowel diseases |
|  | 2\_Lipotoxicity in NAFLD |
|  | 1\_CD4+ T cell and epithelial cell response in celiac disease |
|  | 5\_Human papilloma virus E6 and E7 proteins promote epithelial cell proliferation |
|  | 1\_2\_Feedback relations between coagulation and fibrinolysis |
|  | 4\_3\_Insulin resistance: FFA-related insulin resistance |
|  | 2\_Ion channel CFTR failure in cystic fibrosis airway epithelium (class III-IV mutations) |
|  | 1\_Antigen-presenting cells promote islet dysfunction and immune system activation in T1D |
|  | 2\_Neuron and Schwann cell death |
|  | Figure 4\_Diabetes mellitus Type 1: conventional dendritic cell |
|  | drug-target pairs direct regulation |
|  | Model of PPARG signaling in psoriais (tested) |

---

|  |  |
| --- | --- |
| Group | Genes with Mutations Associated with Atherosclerosis |
|  | Genes Associated with Ulcerative Colitis |
|  | Genes with Polymorphisms Associated with Migraine |
|  | Genes with Susceptibility Loci Associated with Age-Related Macular Degeneration |

---

|  |  |
| --- | --- |
| MedScan ID | 7099 |

---

|  |  |
| --- | --- |
| LocusLink ID | 7099 |
|  | 21898 |
|  | 29260 |

---

|  |  |
| --- | --- |
| Alias | hToll |
|  | age-related macular degeneration 10 |
|  | homolog of toll |
|  | Ly87 |
|  | toll-like receptor 4 |
|  | RP24-209H15.1 |
|  | Ran/M I |
|  | Ran/M1 |
|  | CD284 antigen |
|  | homologue of toll |
|  | CD284 |
|  | OTTHUMP00000022807 |
|  | antigen CD284 |
|  | homolog of Drosophila toll |
|  | TLR4 |
|  | toll4 |
|  | homologue of Drosophila toll |
|  | toll-like receptor IV |
|  | OTTMUSP00000000296 |
|  | ARMD10 |
|  | TOLL |
|  | TLR-4 |
|  | toll like receptor 4 protein |
|  | Lps |
|  | Rasl2-8 |
|  | Toll like receptor 4 |
|  | lipopolysaccharide response |
|  | homolog of toll |
|  | Ran/M1s |

---

|  |  |
| --- | --- |
| GO ID | 0001540 |
|  | 0001530 |
|  | 0001875 |
|  | 0046982 |
|  | 0038023 |
|  | 0005102 |
|  | 0004888 |
|  | 0002322 |
|  | 0007249 |
|  | 0007252 |
|  | 0002755 |
|  | 0002756 |
|  | 0042088 |
|  | 0035666 |
|  | 0000187 |
|  | 0097190 |
|  | 0014002 |
|  | 1904646 |
|  | 0071346 |
|  | 0071222 |
|  | 0071223 |
|  | 0071260 |
|  | 0140052 |
|  | 0050829 |
|  | 0042742 |
|  | 0016046 |
|  | 0032497 |
|  | 0006955 |
|  | 0006954 |
|  | 0045087 |
|  | 0032609 |
|  | 0050702 |
|  | 0060729 |
|  | 0031663 |
|  | 0042116 |
|  | 0070266 |
|  | 0070373 |
|  | 0034128 |
|  | 0120163 |
|  | 0032689 |
|  | 0032700 |
|  | 0032707 |
|  | 0032715 |
|  | 0045671 |
|  | 0032720 |
|  | 0002537 |
|  | 0006909 |
|  | 0030890 |
|  | 0070374 |
|  | 0043123 |
|  | 0046330 |
|  | 0045348 |
|  | 0051092 |
|  | 1901224 |
|  | 1900227 |
|  | 1903974 |
|  | 0032722 |
|  | 1900017 |
|  | 0010628 |
|  | 0050729 |
|  | 0032727 |
|  | 0045359 |
|  | 0032728 |
|  | 0032729 |
|  | 0050718 |
|  | 0032732 |
|  | 0032733 |
|  | 0045084 |
|  | 0032735 |
|  | 0032755 |
|  | 0045416 |
|  | 0032757 |
|  | 0043032 |
|  | 0060907 |
|  | 0045429 |
|  | 0051770 |
|  | 0070430 |
|  | 0070434 |
|  | 1903223 |
|  | 0010572 |
|  | 1903428 |
|  | 0045944 |
|  | 0042535 |
|  | 0032760 |
|  | 0002730 |
|  | 0032496 |
|  | 0034142 |
|  | 0002224 |
|  | 0009986 |
|  | 0005737 |
|  | 0005769 |
|  | 0010008 |
|  | 0009897 |
|  | 0005887 |
|  | 0031226 |
|  | 0046696 |
|  | 0048471 |
|  | 0001891 |
|  | 0005886 |
|  | 0043235 |
|  | 0043548 |
|  | 0007250 |
|  | 0002218 |
|  | 0050966 |
|  | 0050965 |
|  | 0002376 |
|  | 0002758 |
|  | 0006691 |
|  | 0045576 |
|  | 0001774 |
|  | 0043388 |
|  | 0051091 |
|  | 0043065 |
|  | 0045362 |
|  | 0045368 |
|  | 0045410 |
|  | 0050671 |
|  | 0050731 |
|  | 0032874 |
|  | 0050727 |
|  | 0051930 |
|  | 1904467 |
|  | 0009617 |
|  | 0045471 |
|  | 0070542 |
|  | 0006979 |
|  | 0007165 |
|  | 0005768 |
|  | 0016021 |
|  | 0016020 |
|  | 0045121 |
|  | 0071300 |
|  | 0016064 |
|  | 0014823 |
|  | 0051384 |
|  | 1990268 |
|  | 0001666 |
|  | 0032868 |
|  | 0043278 |
|  | 0032570 |
|  | 0032526 |
|  | 0009636 |
|  | 0003009 |
|  | 0005622 |
|  | 0050707 |
|  | 0005515 |
|  | 0008063 |
|  | 0038123 |
|  | 0038124 |
|  | 0004872 |
|  | 0002282 |
|  | 0034134 |
|  | 0034138 |
|  | 0034130 |
|  | 0042346 |
|  | 0052033 |
|  | 0010552 |
|  | 0031224 |
|  | 0070391 |
|  | 0003824 |
|  | 0008152 |
|  | 0016045 |

---

|  |  |
| --- | --- |
| KEGG ID | hsa:7099 |
|  | mmu:21898 |
|  | rno:29260 |

---

|  |  |
| --- | --- |
| MedLine Reference | 10359581 |
|  | 10426995 |
|  | 10524757 |
|  | 10734131 |
|  | 10835634 |
|  | 10952994 |
|  | 11081518 |
|  | 11104518 |
|  | 11254600 |
|  | 11466383 |
|  | 11477091 |
|  | 11490012 |
|  | 11514453 |
|  | 11526399 |
|  | 11544529 |
|  | 11561001 |
|  | 11688988 |
|  | 11706042 |
|  | 11728442 |
|  | 11743586 |
|  | 11751856 |
|  | 11782555 |
|  | 11836257 |
|  | 11841848 |
|  | 11842086 |
|  | 11877429 |
|  | 11893689 |
|  | 11912201 |
|  | 11923281 |
|  | 11932926 |
|  | 11932927 |
|  | 11964313 |
|  | 11970998 |
|  | 11976338 |
|  | 12045108 |
|  | 12055604 |
|  | 12089142 |
|  | 12117914 |
|  | 12124407 |
|  | 12133979 |
|  | 12165534 |
|  | 12174084 |
|  | 12193670 |
|  | 12196527 |
|  | 12270725 |
|  | 12324469 |
|  | 12391239 |
|  | 12397216 |
|  | 12402214 |
|  | 12406828 |
|  | 12411706 |
|  | 12438323 |
|  | 12444143 |
|  | 12477932 |
|  | 12495941 |
|  | 12525572 |
|  | 12588460 |
|  | 12591474 |
|  | 12592402 |
|  | 12594207 |
|  | 12599057 |
|  | 12622779 |
|  | 12630564 |
|  | 12717385 |
|  | 12724322 |
|  | 12730365 |
|  | 12734376 |
|  | 12738639 |
|  | 12742999 |
|  | 12773175 |
|  | 12773319 |
|  | 12777373 |
|  | 12782302 |
|  | 12794153 |
|  | 12796470 |
|  | 12810071 |
|  | 12832426 |
|  | 12846053 |
|  | 12855579 |
|  | 12865424 |
|  | 12874320 |
|  | 12897738 |
|  | 12923493 |
|  | 12925853 |
|  | 12941697 |
|  | 12942028 |
|  | 12957699 |
|  | 12960171 |
|  | 12964127 |
|  | 14517278 |
|  | 14519765 |
|  | 14556004 |
|  | 14557267 |
|  | 14563652 |
|  | 14565959 |
|  | 14578307 |
|  | 14597728 |
|  | 14600154 |
|  | 14610481 |
|  | 14638766 |
|  | 14693986 |
|  | 14699116 |
|  | 14699155 |
|  | 14706103 |
|  | 14709406 |
|  | 14764599 |
|  | 15016407 |
|  | 15022344 |
|  | 15039334 |
|  | 15041961 |
|  | 15044215 |
|  | 15069085 |
|  | 15081257 |
|  | 15107846 |
|  | 15132988 |
|  | 15138173 |
|  | 15148609 |
|  | 15155619 |
|  | 15175334 |
|  | 15187145 |
|  | 15213237 |
|  | 15225640 |
|  | 15247281 |
|  | 15258789 |
|  | 15277575 |
|  | 15305230 |
|  | 15321997 |
|  | 15337750 |
|  | 15340161 |
|  | 15351720 |
|  | 15356101 |
|  | 15356557 |
|  | 15358455 |
|  | 15385480 |
|  | 15452251 |
|  | 15456896 |
|  | 15489631 |
|  | 15509550 |
|  | 15528333 |
|  | 15547160 |
|  | 15557191 |
|  | 15576653 |
|  | 15610077 |
|  | 15614130 |
|  | 15623538 |
|  | 15632890 |
|  | 15644417 |
|  | 15655821 |
|  | 15661045 |
|  | 15665723 |
|  | 15670831 |
|  | 15691841 |
|  | 15694388 |
|  | 15695310 |
|  | 15699327 |
|  | 15728517 |
|  | 15752733 |
|  | 15760452 |
|  | 15760459 |
|  | 15770725 |
|  | 15778388 |
|  | 15790341 |
|  | 15796767 |
|  | 15809303 |
|  | 15829498 |
|  | 15843568 |
|  | 15845500 |
|  | 15852007 |
|  | 15863460 |
|  | 15864121 |
|  | 15866212 |
|  | 15866876 |
|  | 15879290 |
|  | 15883205 |
|  | 15905704 |
|  | 15910421 |
|  | 15910856 |
|  | 15919371 |
|  | 15927851 |
|  | 15932772 |
|  | 15972671 |
|  | 15973118 |
|  | 16002719 |
|  | 16006099 |
|  | 16044857 |
|  | 16135957 |
|  | 16142747 |
|  | 16146574 |
|  | 16157088 |
|  | 16157451 |
|  | 16213463 |
|  | 16215326 |
|  | 16219107 |
|  | 16239565 |
|  | 16266379 |
|  | 16267105 |
|  | 16270639 |
|  | 16290232 |
|  | 16338228 |
|  | 16338476 |
|  | 16343635 |
|  | 16357190 |
|  | 16371473 |
|  | 16397127 |
|  | 16436969 |
|  | 16439361 |
|  | 16443229 |
|  | 16469362 |
|  | 16508260 |
|  | 16517732 |
|  | 16523497 |
|  | 16552356 |
|  | 16556150 |
|  | 16581829 |
|  | 16585591 |
|  | 16603631 |
|  | 16613760 |
|  | 16629651 |
|  | 16651628 |
|  | 16706818 |
|  | 16714144 |
|  | 16717116 |
|  | 16731773 |
|  | 16738534 |
|  | 16751411 |
|  | 16754682 |
|  | 16754684 |
|  | 16754725 |
|  | 16779724 |
|  | 16785528 |
|  | 16786156 |
|  | 16802353 |
|  | 16818785 |
|  | 16829202 |
|  | 16830219 |
|  | 16843693 |
|  | 16879199 |
|  | 16888192 |
|  | 16933467 |
|  | 16950283 |
|  | 16955390 |
|  | 16969132 |
|  | 16973891 |
|  | 16990552 |
|  | 17008589 |
|  | 17015691 |
|  | 17038528 |
|  | 17055575 |
|  | 17056597 |
|  | 17057796 |
|  | 17086894 |
|  | 17096074 |
|  | 17100621 |
|  | 17100625 |
|  | 17121827 |
|  | 17130564 |
|  | 17143969 |
|  | 17151549 |
|  | 17156435 |
|  | 17157899 |
|  | 17172927 |
|  | 17220311 |
|  | 17229603 |
|  | 17237368 |
|  | 17264400 |
|  | 17292969 |
|  | 17301819 |
|  | 17304101 |
|  | 17309585 |
|  | 17314284 |
|  | 17314700 |
|  | 17328955 |
|  | 17332447 |
|  | 17344072 |
|  | 17362744 |
|  | 17373355 |
|  | 17381401 |
|  | 17389503 |
|  | 17390084 |
|  | 17395901 |
|  | 17414051 |
|  | 17431743 |
|  | 17449325 |
|  | 17465679 |
|  | 17493663 |
|  | 17513785 |
|  | 17530716 |
|  | 17538633 |
|  | 17549384 |
|  | 17555409 |
|  | 17556642 |
|  | 17559631 |
|  | 17565323 |
|  | 17579019 |
|  | 17579031 |
|  | 17592301 |
|  | 17597463 |
|  | 17599732 |
|  | 17600225 |
|  | 17675516 |
|  | 17707233 |
|  | 17713436 |
|  | 9237759 |
|  | 9435236 |
|  | 9625770 |
|  | 10047546 |
|  | 10047547 |
|  | 10087992 |
|  | 101590 |
|  | 10201887 |
|  | 10229796 |
|  | 10349636 |
|  | 10358184 |
|  | 10431187 |
|  | 10452977 |
|  | 10500213 |
|  | 10548109 |
|  | 10549626 |
|  | 10607756 |
|  | 10623794 |
|  | 10663571 |
|  | 10669111 |
|  | 10679411 |
|  | 10770809 |
|  | 10783135 |
|  | 1086804 |
|  | 1097575 |
|  | 11042159 |
|  | 11061032 |
|  | 11067888 |
|  | 11069716 |
|  | 11076861 |
|  | 11123338 |
|  | 11123339 |
|  | 11145696 |
|  | 11159012 |
|  | 11171016 |
|  | 11217851 |
|  | 11261784 |
|  | 11286707 |
|  | 11294571 |
|  | 11310845 |
|  | 11313410 |
|  | 11343210 |
|  | 11349049 |
|  | 11489994 |
|  | 11500829 |
|  | 11739561 |
|  | 11748204 |
|  | 11777976 |
|  | 11778656 |
|  | 11801667 |
|  | 11854525 |
|  | 11884465 |
|  | 11923412 |
|  | 11962736 |
|  | 11994480 |
|  | 12003842 |
|  | 12020131 |
|  | 12055204 |
|  | 12055206 |
|  | 12069074 |
|  | 12072368 |
|  | 12165539 |
|  | 12183533 |
|  | 12195388 |
|  | 12218091 |
|  | 12218133 |
|  | 12234819 |
|  | 12244136 |
|  | 12244183 |
|  | 12244198 |
|  | 123543 |
|  | 12354379 |
|  | 12355432 |
|  | 12356687 |
|  | 12370224 |
|  | 12402188 |
|  | 12421970 |
|  | 12424240 |
|  | 1244416 |
|  | 12447441 |
|  | 12466142 |
|  | 12466851 |
|  | 12486107 |
|  | 12515817 |
|  | 12532024 |
|  | 12569171 |
|  | 12616480 |
|  | 12616500 |
|  | 12625509 |
|  | 12640135 |
|  | 12655047 |
|  | 12663667 |
|  | 12665857 |
|  | 12671050 |
|  | 12672079 |
|  | 12682225 |
|  | 12686553 |
|  | 12707317 |
|  | 12707353 |
|  | 12727921 |
|  | 12730691 |
|  | 12782711 |
|  | 12794144 |
|  | 12824464 |
|  | 12825186 |
|  | 12854089 |
|  | 12855817 |
|  | 12864815 |
|  | 12875990 |
|  | 12888452 |
|  | 12890349 |
|  | 12890428 |
|  | 12904319 |
|  | 12917260 |
|  | 12918124 |
|  | 12927920 |
|  | 12960264 |
|  | 12960338 |
|  | 12960345 |
|  | 1373998 |
|  | 1376353 |
|  | 14500530 |
|  | 14517279 |
|  | 14530354 |
|  | 14551216 |
|  | 14561708 |
|  | 14568952 |
|  | 14573621 |
|  | 14579265 |
|  | 14617520 |
|  | 14633605 |
|  | 14634059 |
|  | 14634093 |
|  | 14634130 |
|  | 14634135 |
|  | 14636418 |
|  | 14644093 |
|  | 14657211 |
|  | 14660645 |
|  | 14688095 |
|  | 14688304 |
|  | 14699006 |
|  | 14734763 |
|  | 14739339 |
|  | 14742522 |
|  | 14742577 |
|  | 14744795 |
|  | 14749364 |
|  | 14752288 |
|  | 14764668 |
|  | 14764737 |
|  | 14966433 |
|  | 14970116 |
|  | 14976600 |
|  | 14978024 |
|  | 14978090 |
|  | 14978111 |
|  | 15004173 |
|  | 15004187 |
|  | 15004557 |
|  | 15029200 |
|  | 15034058 |
|  | 15034062 |
|  | 15034070 |
|  | 15039311 |
|  | 15039466 |
|  | 15048726 |
|  | 15072965 |
|  | 15086422 |
|  | 15100369 |
|  | 15128808 |
|  | 15128825 |
|  | 15137944 |
|  | 15143282 |
|  | 15163536 |
|  | 15178705 |
|  | 15208271 |
|  | 15214027 |
|  | 15240691 |
|  | 15240706 |
|  | 15240744 |
|  | 15249654 |
|  | 15260992 |
|  | 15265925 |
|  | 15271679 |
|  | 15271901 |
|  | 15280422 |
|  | 15282190 |
|  | 15322195 |
|  | 15322203 |
|  | 15331330 |
|  | 15353553 |
|  | 15358664 |
|  | 15368304 |
|  | 15369778 |
|  | 15375173 |
|  | 1541912 |
|  | 15458779 |
|  | 15458952 |
|  | 15464243 |
|  | 15475494 |
|  | 15485838 |
|  | 15494520 |
|  | 15501775 |
|  | 15501798 |
|  | 15514106 |
|  | 15522205 |
|  | 15530921 |
|  | 15537384 |
|  | 15538945 |
|  | 15539158 |
|  | 15557205 |
|  | 15557622 |
|  | 1555775 |
|  | 15563689 |
|  | 15585830 |
|  | 15585866 |
|  | 15611251 |
|  | 15618172 |
|  | 15618193 |
|  | 15630134 |
|  | 15634894 |
|  | 15638128 |
|  | 15650774 |
|  | 15654341 |
|  | 15659328 |
|  | 15661922 |
|  | 15664935 |
|  | 15671286 |
|  | 15684036 |
|  | 15695506 |
|  | 15699133 |
|  | 15701712 |
|  | 15716415 |
|  | 15718365 |
|  | 15718493 |
|  | 15731053 |
|  | 15731076 |
|  | 15737196 |
|  | 15738005 |
|  | 15755814 |
|  | 15773898 |
|  | 15778492 |
|  | 15781585 |
|  | 15784548 |
|  | 15784698 |
|  | 15795240 |
|  | 1579857 |
|  | 15809417 |
|  | 15814731 |
|  | 15826931 |
|  | 15849871 |
|  | 15864310 |
|  | 15879150 |
|  | 15896296 |
|  | 15902499 |
|  | 15905502 |
|  | 15905587 |
|  | 15919369 |
|  | 15920727 |
|  | 15944297 |
|  | 15961512 |
|  | 15964841 |
|  | 15967420 |
|  | 15972657 |
|  | 15972679 |
|  | 15972684 |
|  | 15994412 |
|  | 15996225 |
|  | 16001129 |
|  | 16002681 |
|  | 16002687 |
|  | 16002720 |
|  | 16024722 |
|  | 16025156 |
|  | 16034104 |
|  | 16040976 |
|  | 16041030 |
|  | 16045730 |
|  | 16052598 |
|  | 16081822 |
|  | 16093364 |
|  | 16113279 |
|  | 16113324 |
|  | 16113340 |
|  | 16116197 |
|  | 16116224 |
|  | 16141072 |
|  | 16141073 |
|  | 16142239 |
|  | 16143103 |
|  | 16148103 |
|  | 16148139 |
|  | 16154177 |
|  | 16163057 |
|  | 16177320 |
|  | 16186163 |
|  | 16210584 |
|  | 16210672 |
|  | 16236128 |
|  | 16237097 |
|  | 16237099 |
|  | 16239544 |
|  | 16239556 |
|  | 16239576 |
|  | 16244651 |
|  | 16254214 |
|  | 16260493 |
|  | 16272176 |
|  | 16272281 |
|  | 16272291 |
|  | 16272328 |
|  | 16272344 |
|  | 16283355 |
|  | 16284190 |
|  | 16284241 |
|  | 16291660 |
|  | 16292312 |
|  | 16293680 |
|  | 16299309 |
|  | 16299326 |
|  | 16301658 |
|  | 16301676 |
|  | 16309564 |
|  | 16316333 |
|  | 16322526 |
|  | 16323245 |
|  | 16325814 |
|  | 16333033 |
|  | 16339563 |
|  | 16344599 |
|  | 16369019 |
|  | 16391018 |
|  | 16394008 |
|  | 16403908 |
|  | 16413386 |
|  | 16413499 |
|  | 16413927 |
|  | 16424940 |
|  | 16425356 |
|  | 16428731 |
|  | 16439473 |
|  | 16455798 |
|  | 16455994 |
|  | 16460678 |
|  | 16461338 |
|  | 16461458 |
|  | 16467877 |
|  | 16469054 |
|  | 16473828 |
|  | 16481293 |
|  | 16481302 |
|  | 16489357 |
|  | 16493066 |
|  | 16500244 |
|  | 16506285 |
|  | 16507888 |
|  | 16517740 |
|  | 16537813 |
|  | 16547261 |
|  | 16574244 |
|  | 16606831 |
|  | 16609927 |
|  | 16622187 |
|  | 16622195 |
|  | 16622218 |
|  | 16628628 |
|  | 16648192 |
|  | 16651610 |
|  | 16670292 |
|  | 16670307 |
|  | 16670331 |
|  | 16675600 |
|  | 16675630 |
|  | 16688168 |
|  | 16703567 |
|  | 16706798 |
|  | 16709864 |
|  | 16714560 |
|  | 16733795 |
|  | 16738669 |
|  | 16757566 |
|  | 16781673 |
|  | 16782847 |
|  | 16790781 |
|  | 16809643 |
|  | 16814255 |
|  | 16829963 |
|  | 16831874 |
|  | 16836770 |
|  | 16847070 |
|  | 16861639 |
|  | 16863919 |
|  | 16877769 |
|  | 16879256 |
|  | 16886064 |
|  | 16888154 |
|  | 16891416 |
|  | 16895974 |
|  | 16912041 |
|  | 16920968 |
|  | 16926427 |
|  | 16943680 |
|  | 16946018 |
|  | 16951362 |
|  | 16954198 |
|  | 16954393 |
|  | 16963319 |
|  | 16980981 |
|  | 16983331 |
|  | 16984919 |
|  | 17000122 |
|  | 17010424 |
|  | 17034585 |
|  | 17034761 |
|  | 17034774 |
|  | 17035347 |
|  | 17038589 |
|  | 17053832 |
|  | 17053835 |
|  | 17060475 |
|  | 17062605 |
|  | 17065506 |
|  | 17071719 |
|  | 17074859 |
|  | 17082484 |
|  | 17082602 |
|  | 17117477 |
|  | 17118979 |
|  | 17126102 |
|  | 17135446 |
|  | 17151286 |
|  | 17154261 |
|  | 17158890 |
|  | 17163447 |
|  | 17164294 |
|  | 17167049 |
|  | 17173973 |
|  | 17178921 |
|  | 17182102 |
|  | 17187407 |
|  | 17188246 |
|  | 17196865 |
|  | 17202335 |
|  | 17210129 |
|  | 17213198 |
|  | 17233566 |
|  | 17237433 |
|  | 17239439 |
|  | 17255318 |
|  | 17267571 |
|  | 17275357 |
|  | 17277152 |
|  | 17277163 |
|  | 17292937 |
|  | 17296755 |
|  | 17322564 |
|  | 17329566 |
|  | 17339355 |
|  | 17353199 |
|  | 17353291 |
|  | 17362743 |
|  | 17372179 |
|  | 17389381 |
|  | 17400552 |
|  | 17403033 |
|  | 17412915 |
|  | 17417641 |
|  | 17433728 |
|  | 17445776 |
|  | 17450126 |
|  | 17456769 |
|  | 17461416 |
|  | 17474083 |
|  | 17475844 |
|  | 17475888 |
|  | 17478729 |
|  | 17493681 |
|  | 17496895 |
|  | 17507094 |
|  | 17513715 |
|  | 17519423 |
|  | 1752882 |
|  | 17535806 |
|  | 17538624 |
|  | 17560196 |
|  | 17573354 |
|  | 17589347 |
|  | 17591966 |
|  | 17617634 |
|  | 17658277 |
|  | 17693552 |
|  | 17707128 |
|  | 1815711 |
|  | 202651 |
|  | 202969 |
|  | 2272660 |
|  | 2277589 |
|  | 2509612 |
|  | 2572648 |
|  | 2631797 |
|  | 2848174 |
|  | 2998065 |
|  | 325138 |
|  | 326565 |
|  | 326955 |
|  | 333293 |
|  | 3397181 |
|  | 342603 |
|  | 342667 |
|  | 359704 |
|  | 368244 |
|  | 3884708 |
|  | 3886795 |
|  | 478638 |
|  | 4877918 |
|  | 501093 |
|  | 627714 |
|  | 6491291 |
|  | 681749 |
|  | 6985638 |
|  | 7365261 |
|  | 7521367 |
|  | 7622247 |
|  | 7650365 |
|  | 7822029 |
|  | 7829969 |
|  | 7849398 |
|  | 792337 |
|  | 7927709 |
|  | 8037746 |
|  | 8330899 |
|  | 8335946 |
|  | 8428530 |
|  | 8654505 |
|  | 8838309 |
|  | 8890215 |
|  | 8921956 |
|  | 8982246 |
|  | 9043949 |
|  | 9108410 |
|  | 915285 |
|  | 9177560 |
|  | 9321479 |
|  | 9403730 |
|  | 9403834 |
|  | 9851930 |
|  | 9989976 |
|  | 10430608 |
|  | 11994482 |
|  | 12616494 |
|  | 14599981 |
|  | 14962484 |
|  | 15060019 |
|  | 15204789 |
|  | 15334694 |
|  | 15712600 |
|  | 15793310 |
|  | 15841051 |
|  | 15935092 |
|  | 16272348 |
|  | 16286274 |
|  | 16489656 |
|  | 16522744 |
|  | 16543639 |
|  | 16870940 |
|  | 16879219 |
|  | 16954392 |
|  | 17167854 |
|  | 17417665 |
|  | 17446308 |
|  | 17512285 |
|  | 17560208 |

---

|  |  |
| --- | --- |
| Organism | Homo sapiens {Organism urn:agi-taxid:9606} |
|  | Mus musculus {Organism urn:agi-taxid:10090} |
|  | Rattus norvegicus {Organism urn:agi-taxid:10116} |
|  | Homo sapiens |
|  | Mus musculus |
|  | Rattus norvegicus |

---

|  |  |
| --- | --- |
| Mouse chromosome position | 4 34.66 cM |
|  | 4 33.0 cM |

---

|  |  |
| --- | --- |
| OMIM ID | 603030 |
|  | 611488 |
|  | 114500 |
|  | 152430 |

---

|  |  |
| --- | --- |
| Rat chromosome position | 5q24 |

---

|  |  |
| --- | --- |
| Hugo ID | 11850 |
|  | HGNC:11850 |

---

|  |  |
| --- | --- |
| Human chromosome position | 9q33.1 |
|  | 9q32-q33 |

---

|  |  |
| --- | --- |
| Swiss-Prot Accession | O00206 |
|  | O00206.2 |
|  | L0CL36 |
|  | Q9QUK6 |
|  | Q9QUK6.1 |
|  | Q9QX05 |
|  | Q9QX05.1 |
|  | A8K1Y8 |
|  | A9XLP9 |
|  | A9XLQ0 |
|  | A9XLQ1 |
|  | B4E194 |
|  | D1CS52 |
|  | D1CS53 |
|  | Q5VZI8 |
|  | Q5VZI9 |
|  | Q9UK78 |
|  | Q9UM57 |
|  | Q9D691 |
|  | Q9QZF5 |
|  | Q9Z203 |
|  | Q3U4D4 |
|  | Q8K2T5 |

---

|  |  |
| --- | --- |
| GenBank ID | NC\_000009 |
|  | NM\_003266 |
|  | NP\_003257 |
|  | NM\_138554 |
|  | NP\_612564 |
|  | NM\_138557 |
|  | NP\_612567 |
|  | NG\_011475 |
|  | AF177765 |
|  | AAF05316 |
|  | AF177766 |
|  | AAF07823 |
|  | AH009665 |
|  | AAF89753 |
|  | AL160272 |
|  | CH471090 |
|  | EAW87448 |
|  | EAW87449 |
|  | EAW87451 |
|  | DQ018107 |
|  | AAY82267 |
|  | DQ018108 |
|  | AAY82268 |
|  | DQ018109 |
|  | AAY82269 |
|  | DQ018110 |
|  | AAY82270 |
|  | EF535829 |
|  | EF535830 |
|  | EF535831 |
|  | ABU41662 |
|  | EF535832 |
|  | ABU41663 |
|  | EF535833 |
|  | ABU41664 |
|  | FB700885 |
|  | CAS02255 |
|  | GM869034 |
|  | CAT16978 |
|  | GQ903698 |
|  | ACX46980 |
|  | GQ903699 |
|  | ACX46981 |
|  | GQ903700 |
|  | ACX46982 |
|  | GQ903701 |
|  | ACX46983 |
|  | GQ903702 |
|  | ACX46984 |
|  | GU071276 |
|  | GU143417 |
|  | ACZ69536 |
|  | GU143418 |
|  | GU143419 |
|  | ACZ69537 |
|  | GU143420 |
|  | ACZ69538 |
|  | GU143421 |
|  | ACZ69539 |
|  | GU143423 |
|  | GU143424 |
|  | ACZ69540 |
|  | GU143425 |
|  | ACZ69541 |
|  | GU143426 |
|  | ACZ69542 |
|  | GU143427 |
|  | ACZ69543 |
|  | JQ480853 |
|  | AFX71929 |
|  | JQ480854 |
|  | AFX71930 |
|  | JQ480855 |
|  | AFX71931 |
|  | JQ480856 |
|  | AFX71932 |
|  | JQ480857 |
|  | AFX71933 |
|  | JQ480858 |
|  | AFX71934 |
|  | JQ480859 |
|  | AFX71935 |
|  | JQ480860 |
|  | AFX71936 |
|  | JQ480861 |
|  | AFX71937 |
|  | JQ480862 |
|  | AFX71938 |
|  | JQ480863 |
|  | AFX71939 |
|  | JQ480864 |
|  | AFX71940 |
|  | JQ480865 |
|  | AFX71941 |
|  | JQ480866 |
|  | AFX71942 |
|  | JQ480867 |
|  | AFX71943 |
|  | JQ480868 |
|  | AFX71944 |
|  | JQ480869 |
|  | AFX71945 |
|  | JQ480870 |
|  | AFX71946 |
|  | JQ480871 |
|  | AFX71947 |
|  | JQ480872 |
|  | AFX71948 |
|  | JQ480873 |
|  | AFX71949 |
|  | JQ480874 |
|  | AFX71950 |
|  | JQ480875 |
|  | AFX71951 |
|  | JQ480876 |
|  | AFX71952 |
|  | JQ480877 |
|  | AFX71953 |
|  | JQ480878 |
|  | AFX71954 |
|  | JQ480879 |
|  | AFX71955 |
|  | JQ480880 |
|  | AFX71956 |
|  | JQ480881 |
|  | AFX71957 |
|  | JQ480882 |
|  | AFX71958 |
|  | JQ480883 |
|  | AFX71959 |
|  | JQ480884 |
|  | AFX71960 |
|  | JQ480885 |
|  | AFX71961 |
|  | JQ480886 |
|  | AFX71962 |
|  | JQ480887 |
|  | AFX71963 |
|  | JQ480888 |
|  | AFX71964 |
|  | JQ480889 |
|  | AFX71965 |
|  | JQ480890 |
|  | AFX71966 |
|  | JQ480891 |
|  | AFX71967 |
|  | JQ480892 |
|  | AFX71968 |
|  | JQ480893 |
|  | AFX71969 |
|  | JQ480894 |
|  | AFX71970 |
|  | JQ480895 |
|  | AFX71971 |
|  | JQ480896 |
|  | AFX71972 |
|  | JQ480897 |
|  | AFX71973 |
|  | JQ480898 |
|  | AFX71974 |
|  | JQ480899 |
|  | AFX71975 |
|  | JQ480900 |
|  | AFX71976 |
|  | JQ480901 |
|  | AFX71977 |
|  | JQ480902 |
|  | AFX71978 |
|  | JQ480903 |
|  | AFX71979 |
|  | JQ480904 |
|  | AFX71980 |
|  | JQ480905 |
|  | AFX71981 |
|  | JQ480906 |
|  | AFX71982 |
|  | JQ480907 |
|  | AFX71983 |
|  | JQ480908 |
|  | AFX71984 |
|  | JQ480909 |
|  | AFX71985 |
|  | JQ480910 |
|  | AFX71986 |
|  | JQ480911 |
|  | AFX71987 |
|  | JQ480912 |
|  | AFX71988 |
|  | JQ480913 |
|  | AFX71989 |
|  | JQ480914 |
|  | AFX71990 |
|  | JQ480915 |
|  | AFX71991 |
|  | JQ480916 |
|  | AFX71992 |
|  | JQ480917 |
|  | AFX71993 |
|  | JQ480918 |
|  | AFX71994 |
|  | JQ480919 |
|  | AFX71995 |
|  | JQ480920 |
|  | AFX71996 |
|  | JQ480921 |
|  | AFX71997 |
|  | JQ480922 |
|  | AFX71998 |
|  | JQ480923 |
|  | AFX71999 |
|  | JQ480924 |
|  | AFX72000 |
|  | JQ480925 |
|  | AFX72001 |
|  | JQ480926 |
|  | AFX72002 |
|  | JQ480927 |
|  | AFX72003 |
|  | JQ480928 |
|  | AFX72004 |
|  | JQ480929 |
|  | AFX72005 |
|  | JQ480930 |
|  | AFX72006 |
|  | JQ480931 |
|  | AFX72007 |
|  | JQ480932 |
|  | AFX72008 |
|  | JQ480933 |
|  | AFX72009 |
|  | JQ480934 |
|  | AFX72010 |
|  | JQ480935 |
|  | AFX72011 |
|  | JQ480936 |
|  | AFX72012 |
|  | JQ480937 |
|  | AFX72013 |
|  | JQ480938 |
|  | AFX72014 |
|  | JQ480939 |
|  | AFX72015 |
|  | JQ480940 |
|  | AFX72016 |
|  | JQ480941 |
|  | AFX72017 |
|  | JQ480942 |
|  | AFX72018 |
|  | JQ480943 |
|  | AFX72019 |
|  | JQ480944 |
|  | AFX72020 |
|  | JQ480945 |
|  | AFX72021 |
|  | JQ480946 |
|  | AFX72022 |
|  | JQ480947 |
|  | AFX72023 |
|  | JQ480948 |
|  | AFX72024 |
|  | JQ480949 |
|  | AFX72025 |
|  | JQ480950 |
|  | AFX72026 |
|  | JQ480951 |
|  | AFX72027 |
|  | JQ480952 |
|  | AFX72028 |
|  | JQ480953 |
|  | AFX72029 |
|  | JQ480954 |
|  | AFX72030 |
|  | JQ480955 |
|  | AFX72031 |
|  | JQ480956 |
|  | AFX72032 |
|  | JQ480957 |
|  | AFX72033 |
|  | JQ480958 |
|  | AFX72034 |
|  | JQ480959 |
|  | AFX72035 |
|  | JQ480960 |
|  | AFX72036 |
|  | JQ480961 |
|  | AFX72037 |
|  | JQ480962 |
|  | AFX72038 |
|  | JQ480963 |
|  | AFX72039 |
|  | JQ480964 |
|  | AFX72040 |
|  | JQ480965 |
|  | AFX72041 |
|  | JQ480966 |
|  | AFX72042 |
|  | JQ480967 |
|  | AFX72043 |
|  | JQ480968 |
|  | AFX72044 |
|  | JQ480969 |
|  | AFX72045 |
|  | JQ480970 |
|  | AFX72046 |
|  | JQ480971 |
|  | AFX72047 |
|  | JQ480972 |
|  | AFX72048 |
|  | JQ480973 |
|  | AFX72049 |
|  | JQ480974 |
|  | AFX72050 |
|  | JQ480975 |
|  | AFX72051 |
|  | JQ480976 |
|  | AFX72052 |
|  | JQ480977 |
|  | AFX72053 |
|  | JQ480978 |
|  | AFX72054 |
|  | JQ480979 |
|  | AFX72055 |
|  | JQ480980 |
|  | AFX72056 |
|  | JQ480981 |
|  | AFX72057 |
|  | JQ480982 |
|  | AFX72058 |
|  | JQ480983 |
|  | AFX72059 |
|  | JQ480984 |
|  | AFX72060 |
|  | JQ480985 |
|  | AFX72061 |
|  | JQ480986 |
|  | AFX72062 |
|  | JQ480987 |
|  | AFX72063 |
|  | JQ480988 |
|  | AFX72064 |
|  | JQ480989 |
|  | AFX72065 |
|  | JQ480990 |
|  | AFX72066 |
|  | JQ480991 |
|  | AFX72067 |
|  | JQ480992 |
|  | AFX72068 |
|  | JQ480993 |
|  | AFX72069 |
|  | JQ480994 |
|  | AFX72070 |
|  | JQ480995 |
|  | AFX72071 |
|  | JQ480996 |
|  | AFX72072 |
|  | JQ480997 |
|  | AFX72073 |
|  | JQ480998 |
|  | AFX72074 |
|  | JQ480999 |
|  | AFX72075 |
|  | JQ481000 |
|  | AFX72076 |
|  | JQ481001 |
|  | AFX72077 |
|  | JQ481002 |
|  | AFX72078 |
|  | JQ481003 |
|  | AFX72079 |
|  | JQ481004 |
|  | AFX72080 |
|  | JQ481005 |
|  | AFX72081 |
|  | JQ481006 |
|  | AFX72082 |
|  | JQ481007 |
|  | AFX72083 |
|  | JQ481008 |
|  | AFX72084 |
|  | JQ481009 |
|  | AFX72085 |
|  | JQ481010 |
|  | AFX72086 |
|  | JQ481011 |
|  | AFX72087 |
|  | JQ481012 |
|  | AFX72088 |
|  | JQ481013 |
|  | AFX72089 |
|  | JQ481014 |
|  | AFX72090 |
|  | JQ481015 |
|  | AFX72091 |
|  | JQ481016 |
|  | AFX72092 |
|  | JQ481017 |
|  | AFX72093 |
|  | JQ481018 |
|  | AFX72094 |
|  | JQ481019 |
|  | AFX72095 |
|  | JQ481020 |
|  | AFX72096 |
|  | JQ481021 |
|  | AFX72097 |
|  | JQ481022 |
|  | AFX72098 |
|  | JQ481023 |
|  | AFX72099 |
|  | JQ481024 |
|  | AFX72100 |
|  | JQ481025 |
|  | AFX72101 |
|  | JQ481026 |
|  | AFX72102 |
|  | JQ481027 |
|  | AFX72103 |
|  | JQ481028 |
|  | AFX72104 |
|  | JQ481029 |
|  | AFX72105 |
|  | JQ481030 |
|  | AFX72106 |
|  | JQ481031 |
|  | AFX72107 |
|  | JQ481032 |
|  | AFX72108 |
|  | JQ481033 |
|  | AFX72109 |
|  | JQ481034 |
|  | AFX72110 |
|  | JQ481035 |
|  | AFX72111 |
|  | JQ481036 |
|  | AFX72112 |
|  | JQ481037 |
|  | AFX72113 |
|  | JQ481038 |
|  | AFX72114 |
|  | JQ481039 |
|  | AFX72115 |
|  | JQ481040 |
|  | AFX72116 |
|  | JQ481041 |
|  | AFX72117 |
|  | JQ481042 |
|  | AFX72118 |
|  | JQ481043 |
|  | AFX72119 |
|  | JQ481044 |
|  | AFX72120 |
|  | JQ481045 |
|  | AFX72121 |
|  | JQ481046 |
|  | AFX72122 |
|  | JQ481047 |
|  | AFX72123 |
|  | JQ481048 |
|  | AFX72124 |
|  | JQ481049 |
|  | AFX72125 |
|  | JQ481050 |
|  | AFX72126 |
|  | JQ481051 |
|  | AFX72127 |
|  | JQ481052 |
|  | AFX72128 |
|  | JQ481053 |
|  | AFX72129 |
|  | JQ481054 |
|  | AFX72130 |
|  | JQ481055 |
|  | AFX72131 |
|  | JQ481056 |
|  | AFX72132 |
|  | JQ481057 |
|  | AFX72133 |
|  | JQ481058 |
|  | AFX72134 |
|  | JQ481059 |
|  | AFX72135 |
|  | JQ481060 |
|  | AFX72136 |
|  | JQ481061 |
|  | AFX72137 |
|  | JQ481062 |
|  | AFX72138 |
|  | JQ481063 |
|  | AFX72139 |
|  | JQ481064 |
|  | AFX72140 |
|  | JQ481065 |
|  | AFX72141 |
|  | JQ481066 |
|  | AFX72142 |
|  | JQ481067 |
|  | AFX72143 |
|  | JQ481068 |
|  | AFX72144 |
|  | JQ481069 |
|  | AFX72145 |
|  | JQ481070 |
|  | AFX72146 |
|  | JQ481071 |
|  | AFX72147 |
|  | JQ481072 |
|  | AFX72148 |
|  | JQ481073 |
|  | AFX72149 |
|  | JQ481074 |
|  | AFX72150 |
|  | JQ481075 |
|  | AFX72151 |
|  | JQ481076 |
|  | AFX72152 |
|  | JQ481077 |
|  | AFX72153 |
|  | JQ481078 |
|  | AFX72154 |
|  | JQ481079 |
|  | AFX72155 |
|  | JQ481080 |
|  | AFX72156 |
|  | JQ481081 |
|  | AFX72157 |
|  | JQ481082 |
|  | AFX72158 |
|  | JQ481083 |
|  | AFX72159 |
|  | JQ481084 |
|  | AFX72160 |
|  | JQ481085 |
|  | AFX72161 |
|  | JQ481086 |
|  | AFX72162 |
|  | JQ481087 |
|  | AFX72163 |
|  | JQ481088 |
|  | AFX72164 |
|  | JQ481089 |
|  | AFX72165 |
|  | JQ481090 |
|  | AFX72166 |
|  | JQ481091 |
|  | AFX72167 |
|  | JQ481092 |
|  | AFX72168 |
|  | JQ481093 |
|  | AFX72169 |
|  | JQ481094 |
|  | AFX72170 |
|  | JQ481095 |
|  | AFX72171 |
|  | JQ481096 |
|  | AFX72172 |
|  | JQ481097 |
|  | AFX72173 |
|  | JQ481098 |
|  | AFX72174 |
|  | JQ481099 |
|  | AFX72175 |
|  | JQ481100 |
|  | AFX72176 |
|  | JQ481101 |
|  | AFX72177 |
|  | JQ481102 |
|  | AFX72178 |
|  | JQ481103 |
|  | AFX72179 |
|  | JQ481104 |
|  | AFX72180 |
|  | JQ481105 |
|  | AFX72181 |
|  | JQ481106 |
|  | AFX72182 |
|  | JQ481107 |
|  | AFX72183 |
|  | JQ481108 |
|  | AFX72184 |
|  | JQ481109 |
|  | AFX72185 |
|  | JQ481110 |
|  | AFX72186 |
|  | JQ481111 |
|  | AFX72187 |
|  | JQ481112 |
|  | AFX72188 |
|  | JQ481113 |
|  | AFX72189 |
|  | JQ481114 |
|  | AFX72190 |
|  | JQ481115 |
|  | AFX72191 |
|  | JQ481116 |
|  | AFX72192 |
|  | JQ481117 |
|  | AFX72193 |
|  | JQ481118 |
|  | AFX72194 |
|  | JQ481119 |
|  | AFX72195 |
|  | JQ481120 |
|  | AFX72196 |
|  | JQ481121 |
|  | AFX72197 |
|  | JQ481122 |
|  | AFX72198 |
|  | JQ481123 |
|  | AFX72199 |
|  | JQ481124 |
|  | AFX72200 |
|  | JQ481125 |
|  | AFX72201 |
|  | JQ481126 |
|  | AFX72202 |
|  | JQ481127 |
|  | AFX72203 |
|  | JQ481128 |
|  | AFX72204 |
|  | JQ481129 |
|  | AFX72205 |
|  | JQ481130 |
|  | AFX72206 |
|  | JQ481131 |
|  | AFX72207 |
|  | JQ481132 |
|  | AFX72208 |
|  | JQ481133 |
|  | AFX72209 |
|  | JQ481134 |
|  | AFX72210 |
|  | JQ481135 |
|  | AFX72211 |
|  | JQ481136 |
|  | AFX72212 |
|  | JQ481137 |
|  | AFX72213 |
|  | JQ481138 |
|  | AFX72214 |
|  | JQ481139 |
|  | AFX72215 |
|  | JQ481140 |
|  | AFX72216 |
|  | JQ481141 |
|  | AFX72217 |
|  | JQ481142 |
|  | AFX72218 |
|  | JQ481143 |
|  | AFX72219 |
|  | JQ481144 |
|  | AFX72220 |
|  | JQ481145 |
|  | AFX72221 |
|  | JQ481146 |
|  | AFX72222 |
|  | JQ481147 |
|  | AFX72223 |
|  | JQ481148 |
|  | AFX72224 |
|  | JQ481149 |
|  | AFX72225 |
|  | JQ481150 |
|  | AFX72226 |
|  | JQ481151 |
|  | AFX72227 |
|  | JQ481152 |
|  | AFX72228 |
|  | JQ481153 |
|  | AFX72229 |
|  | JQ481154 |
|  | AFX72230 |
|  | JQ481155 |
|  | AFX72231 |
|  | JQ481156 |
|  | AFX72232 |
|  | JQ481157 |
|  | AFX72233 |
|  | JQ481158 |
|  | AFX72234 |
|  | JQ481159 |
|  | AFX72235 |
|  | JQ481160 |
|  | AFX72236 |
|  | JQ481161 |
|  | AFX72237 |
|  | JQ481162 |
|  | AFX72238 |
|  | JQ481163 |
|  | AFX72239 |
|  | JQ481164 |
|  | AFX72240 |
|  | JQ481165 |
|  | AFX72241 |
|  | JQ481166 |
|  | AFX72242 |
|  | JQ481167 |
|  | AFX72243 |
|  | JQ481168 |
|  | AFX72244 |
|  | JQ481169 |
|  | AFX72245 |
|  | JQ481170 |
|  | AFX72246 |
|  | JQ481171 |
|  | AFX72247 |
|  | JQ481172 |
|  | AFX72248 |
|  | JQ481173 |
|  | AFX72249 |
|  | JQ481174 |
|  | AFX72250 |
|  | JQ481175 |
|  | AFX72251 |
|  | JQ481176 |
|  | AFX72252 |
|  | JQ481177 |
|  | AFX72253 |
|  | JQ481178 |
|  | AFX72254 |
|  | JQ481179 |
|  | AFX72255 |
|  | JQ481180 |
|  | AFX72256 |
|  | JQ481181 |
|  | AFX72257 |
|  | JQ481182 |
|  | AFX72258 |
|  | JQ481183 |
|  | AFX72259 |
|  | JQ481184 |
|  | AFX72260 |
|  | JQ481185 |
|  | AFX72261 |
|  | JQ481186 |
|  | AFX72262 |
|  | JQ481187 |
|  | AFX72263 |
|  | JQ481188 |
|  | AFX72264 |
|  | JQ481189 |
|  | AFX72265 |
|  | JQ481190 |
|  | AFX72266 |
|  | JQ481191 |
|  | AFX72267 |
|  | JQ481192 |
|  | AFX72268 |
|  | JQ481193 |
|  | AFX72269 |
|  | JQ481194 |
|  | AFX72270 |
|  | JQ481195 |
|  | AFX72271 |
|  | JQ481196 |
|  | AFX72272 |
|  | JQ481197 |
|  | AFX72273 |
|  | JQ481198 |
|  | AFX72274 |
|  | JQ481199 |
|  | AFX72275 |
|  | JQ481200 |
|  | AFX72276 |
|  | JQ481201 |
|  | AFX72277 |
|  | JQ481202 |
|  | AFX72278 |
|  | JQ481203 |
|  | AFX72279 |
|  | JQ481204 |
|  | AFX72280 |
|  | JQ481205 |
|  | AFX72281 |
|  | JQ481206 |
|  | AFX72282 |
|  | JQ481207 |
|  | AFX72283 |
|  | JQ481208 |
|  | AFX72284 |
|  | JQ481209 |
|  | AFX72285 |
|  | JQ481210 |
|  | AFX72286 |
|  | JQ481211 |
|  | AFX72287 |
|  | JQ481212 |
|  | AFX72288 |
|  | JQ481213 |
|  | AFX72289 |
|  | JQ481214 |
|  | AFX72290 |
|  | JQ481215 |
|  | AFX72291 |
|  | JQ481216 |
|  | AFX72292 |
|  | JQ481217 |
|  | AFX72293 |
|  | JQ481218 |
|  | AFX72294 |
|  | JQ481219 |
|  | AFX72295 |
|  | JQ481220 |
|  | AFX72296 |
|  | JQ481221 |
|  | AFX72297 |
|  | JQ481222 |
|  | AFX72298 |
|  | JQ481223 |
|  | AFX72299 |
|  | JQ481224 |
|  | AFX72300 |
|  | JQ481225 |
|  | AFX72301 |
|  | JQ481226 |
|  | AFX72302 |
|  | JQ481227 |
|  | AFX72303 |
|  | JQ481228 |
|  | AFX72304 |
|  | JQ481229 |
|  | AFX72305 |
|  | JQ481230 |
|  | AFX72306 |
|  | JQ481231 |
|  | AFX72307 |
|  | JQ481232 |
|  | AFX72308 |
|  | JQ481233 |
|  | AFX72309 |
|  | JQ481234 |
|  | AFX72310 |
|  | JQ481235 |
|  | AFX72311 |
|  | JQ481236 |
|  | AFX72312 |
|  | JQ481237 |
|  | AFX72313 |
|  | JQ481238 |
|  | AFX72314 |
|  | JQ481239 |
|  | AFX72315 |
|  | JQ481240 |
|  | AFX72316 |
|  | JQ481241 |
|  | AFX72317 |
|  | MG385628 |
|  | AZI70866 |
|  | AA598398 |
|  | AB445638 |
|  | BAG55035 |
|  | AK226155 |
|  | AK290053 |
|  | BAF82742 |
|  | AK293068 |
|  | BAF85757 |
|  | AK303730 |
|  | BAG64706 |
|  | AL710219 |
|  | BC025294 |
|  | BC117422 |
|  | AAI17423 |
|  | BC143841 |
|  | BG622357 |
|  | DA126770 |
|  | DA187988 |
|  | DA218006 |
|  | U69193 |
|  | U88880 |
|  | AAC34135 |
|  | U93091 |
|  | AAC80227 |
|  | O00206 |
|  | NC\_000070 |
|  | NM\_021297 |
|  | NP\_067272 |
|  | AF177767 |
|  | AAF05317 |
|  | AL805946 |
|  | CAD83014 |
|  | BX649609 |
|  | CH466527 |
|  | EDL31078 |
|  | KC811649 |
|  | AGX25444 |
|  | KC811650 |
|  | AGX25445 |
|  | KC811651 |
|  | AGX25446 |
|  | KC811652 |
|  | AGX25447 |
|  | KC811653 |
|  | AGX25448 |
|  | KC811654 |
|  | AGX25449 |
|  | KC811655 |
|  | AGX25450 |
|  | KC811656 |
|  | AGX25451 |
|  | KC811657 |
|  | AGX25452 |
|  | KF696867 |
|  | AGY29656 |
|  | KF696868 |
|  | AGY29657 |
|  | KF696869 |
|  | AGY29658 |
|  | KF696870 |
|  | AGY29659 |
|  | KF696871 |
|  | AGY29660 |
|  | KF696872 |
|  | AGY29661 |
|  | KF696873 |
|  | AGY29662 |
|  | KF696874 |
|  | AGY29663 |
|  | KF696875 |
|  | AGY29664 |
|  | KF696876 |
|  | AGY29665 |
|  | KF696877 |
|  | AGY29666 |
|  | KF696878 |
|  | AGY29667 |
|  | KF696879 |
|  | AGY29668 |
|  | KF696880 |
|  | AGY29669 |
|  | KF696881 |
|  | AGY29670 |
|  | KF696882 |
|  | AGY29671 |
|  | KF696883 |
|  | AGY29672 |
|  | KF696884 |
|  | AGY29673 |
|  | KF696885 |
|  | AGY29674 |
|  | KF696886 |
|  | AGY29675 |
|  | KF696887 |
|  | AGY29676 |
|  | KF696888 |
|  | AGY29677 |
|  | KF696889 |
|  | AGY29678 |
|  | KF696890 |
|  | AGY29679 |
|  | KF696891 |
|  | AGY29680 |
|  | KF696892 |
|  | AGY29681 |
|  | KF696893 |
|  | AGY29682 |
|  | KF696894 |
|  | AGY29683 |
|  | KF696895 |
|  | AGY29684 |
|  | KF696896 |
|  | AGY29685 |
|  | KF696897 |
|  | AGY29686 |
|  | KF696898 |
|  | AGY29687 |
|  | KF696899 |
|  | AGY29688 |
|  | KF696900 |
|  | AGY29689 |
|  | KF696901 |
|  | AGY29690 |
|  | KF696902 |
|  | AGY29691 |
|  | KF696903 |
|  | AGY29692 |
|  | KF696904 |
|  | AGY29693 |
|  | KF696905 |
|  | AGY29694 |
|  | KF696906 |
|  | AGY29695 |
|  | KF696907 |
|  | AGY29696 |
|  | KF696908 |
|  | AGY29697 |
|  | KF696909 |
|  | AGY29698 |
|  | KF696910 |
|  | AGY29699 |
|  | KF696911 |
|  | AGY29700 |
|  | KF696912 |
|  | AGY29701 |
|  | KF696913 |
|  | AGY29702 |
|  | KF696914 |
|  | AGY29703 |
|  | KF696915 |
|  | AGY29704 |
|  | KF696916 |
|  | AGY29705 |
|  | KF696917 |
|  | AGY29706 |
|  | KF696918 |
|  | AGY29707 |
|  | KF696919 |
|  | AGY29708 |
|  | KF696920 |
|  | AGY29709 |
|  | KF696921 |
|  | AGY29710 |
|  | KF696922 |
|  | AGY29711 |
|  | KF696923 |
|  | AGY29712 |
|  | KF696924 |
|  | AGY29713 |
|  | KF696925 |
|  | AGY29714 |
|  | KF696926 |
|  | AGY29715 |
|  | KF696927 |
|  | AGY29716 |
|  | KF696928 |
|  | AGY29717 |
|  | KF696929 |
|  | AGY29718 |
|  | KF696930 |
|  | AGY29719 |
|  | KF696931 |
|  | AGY29720 |
|  | KF696932 |
|  | AGY29721 |
|  | KF696933 |
|  | AGY29722 |
|  | KF696934 |
|  | AGY29723 |
|  | KF696935 |
|  | AGY29724 |
|  | KF696936 |
|  | AGY29725 |
|  | KF696937 |
|  | AGY29726 |
|  | KF696938 |
|  | AGY29727 |
|  | KF696939 |
|  | AGY29728 |
|  | KF696940 |
|  | AGY29729 |
|  | KF696941 |
|  | AGY29730 |
|  | KF696942 |
|  | AGY29731 |
|  | KF696943 |
|  | AGY29732 |
|  | KF696944 |
|  | AGY29733 |
|  | KF696945 |
|  | AGY29734 |
|  | KF696946 |
|  | AGY29735 |
|  | KF696947 |
|  | AGY29736 |
|  | KF696948 |
|  | AGY29737 |
|  | KF696949 |
|  | AGY29738 |
|  | KF696950 |
|  | AGY29739 |
|  | KF696951 |
|  | AGY29740 |
|  | KF696952 |
|  | AGY29741 |
|  | KF696953 |
|  | AGY29742 |
|  | KF696954 |
|  | AGY29743 |
|  | KF696955 |
|  | AGY29744 |
|  | KF696956 |
|  | AGY29745 |
|  | KF696957 |
|  | AGY29746 |
|  | KF696958 |
|  | AGY29747 |
|  | KF696959 |
|  | AGY29748 |
|  | KF696960 |
|  | AGY29749 |
|  | KF696961 |
|  | AGY29750 |
|  | KF696962 |
|  | AGY29751 |
|  | KF696963 |
|  | AGY29752 |
|  | KF696964 |
|  | AGY29753 |
|  | KF696965 |
|  | AGY29754 |
|  | KF696966 |
|  | AGY29755 |
|  | KF696967 |
|  | AGY29756 |
|  | AF095353 |
|  | AAC99411 |
|  | AF110133 |
|  | AAD29272 |
|  | AF185285 |
|  | AAF04278 |
|  | AI450864 |
|  | AK014533 |
|  | AK030348 |
|  | AK154297 |
|  | BAE32498 |
|  | AK170566 |
|  | BC029856 |
|  | AAH29856 |
|  | BX514079 |
|  | CD544295 |
|  | JX878358 |
|  | AGA16685 |
|  | JX878359 |
|  | AGA16686 |
|  | Q9QUK6 |
|  | NC\_005104 |
|  | NM\_019178 |
|  | NP\_062051 |
|  | AC\_000073 |
|  | AABR07048553 |
|  | AAHX01037335 |
|  | CH473978 |
|  | EDM10505 |
|  | AF057025 |
|  | AAC13313 |
|  | EU929065 |
|  | ACI15385 |
|  | FQ233814 |
|  | GQ175880 |
|  | ACS45118 |
|  | Q9QX05 |
|  | AF172171 |
|  | XP\_005252239 |
|  | CAH72619 |
|  | CAH72618 |
|  | AC\_000141 |
|  | XM\_005252182 |
|  | AMYH02021544 |
|  | CAI26152 |
|  | AABR06037636 |
|  | AAHY01036550 |
|  | CAM27682 |
|  | AC\_000026 |
|  | NC\_018920 |
|  | ABBA01016273 |
|  | NR\_024168 |
|  | NR\_024169 |
|  | NT\_008470 |
|  | NW\_001839237 |
|  | NW\_924573 |
|  | AC\_000052 |
|  | NT\_039260 |
|  | NW\_001030747 |
|  | Q3U4D4 |
|  | Q8K2T5 |
|  | NW\_001084840 |
|  | NW\_047714 |
|  | NT\_039288 |
|  | 109659094 |
|  | 119607857 |
|  | 20140413 |
|  | 2429103 |
|  | 2459628 |
|  | 55662033 |
|  | 55662034 |
|  | 6175873 |
|  | 6403467 |
|  | 74747809 |
|  | 74747810 |
|  | 74747811 |
|  | 9622357 |
|  | Q5VZI7 |
|  | Q5VZI8 |
|  | Q5VZI9 |
|  | 123784217 |
|  | 148699131 |
|  | 20140894 |
|  | 20988279 |
|  | 4092077 |
|  | 4761197 |
|  | 56238167 |
|  | 6120054 |
|  | 6175875 |
|  | 74178484 |
|  | 81870258 |
|  | 81878581 |
|  | AV230010 |
|  | Q5RGT4 |
|  | 149059567 |
|  | 20140357 |
|  | 3044208 |

---

|  |  |
| --- | --- |
| Swiss-Prot ID | TLR4\_HUMAN |
|  | TLR4\_MOUSE |
|  | TLR4\_RAT |
|  | D1CS53\_HUMAN |

---

|  |  |
| --- | --- |
| Cell Localization | Cell membrane |
|  | Early endosome |
|  | Membrane |
|  | Plasma membrane |

---

|  |  |
| --- | --- |
| Ensembl ID | ENSG00000136869 |
|  | ENSP00000377997.4 |
|  | ENST00000394487.5 |
|  | ENSP00000494590.1 |
|  | ENST00000645071.1 |
|  | ENSMUSG00000039005 |
|  | ENSMUSP00000045770.5 |
|  | ENSMUST00000048096.11 |
|  | ENSRNOG00000010522 |
|  | ENSRNOP00000014020.2 |
|  | ENSRNOT00000014020.3 |
|  | ENSRNOT00000014020 |
|  | ENSP00000363089 |
|  | ENSP00000363089.5 |
|  | ENST00000355622 |
|  | ENSP00000377997 |
|  | ENSMUST00000048096 |
|  | ENST00000355622.7 |
|  | ENST00000394487 |
|  | ENSRNOP00000014020 |
|  | ENSMUSP00000045770 |

---

|  |  |
| --- | --- |
| MGI ID | MGI:96824 |
|  | 96824 |

---

|  |  |
| --- | --- |
| RGD ID | 3870 |

---

|  |  |
| --- | --- |
| TCDB ID | 8.A.43.1.9 |

---

|  |  |
| --- | --- |
| Unigene ID | Rn.14534 |
|  | Hs.174312 |
|  | Mm.38049 |

---

|  |  |
| --- | --- |
| Homologene ID | 41317 |

---

|  |  |
| --- | --- |
| Shape | Stick-vertex |

---

|  |  |
| --- | --- |
| IPI ID | IPI00212570 |
|  | IPI00131730 |
|  | IPI00914904 |
|  | IPI00008673 |
|  | IPI00374265 |
|  | IPI00649472 |

---

|  |  |
| --- | --- |
| Homo sapiens Chromosome position | 9q32-q33 |

---

|  |  |
| --- | --- |
| Ensembl Transcript ID | ENST00000355622 |

---

|  |  |
| --- | --- |
| FunctionalClass | lipopolysaccharide binding |
|  | protein binding |
|  | transmembrane receptor |
|  | catalytic |
|  | receptor |
|  | phosphoinositide 3-kinase binding |

---

|  |  |
| --- | --- |
| Mus musculus Chromosome position | 4 33.0 cM |

---

|  |  |
| --- | --- |
| KEGG pathway | Toll-like receptor signaling pathway |

---

|  |  |
| --- | --- |
| Rattus norvegicus Chromosome position | 5q24 |

---

|  |  |
| --- | --- |
| Ensembl Gene ID | ENSG00000136869 |

---
